# Preliminary Investigation on the Relationship between Raman Spectra of Beef and Metmyoglobin and Metmyoglobin Reductase Activity

**DOI:** 10.1155/2022/4117261

**Published:** 2022-10-13

**Authors:** Tonggang Zhang, Yalei Li, Ruiming Luo, Shuang Bo

**Affiliations:** ^1^School of Biology and Brewing Engineering, Taishan University, Shandong 271000, China; ^2^School of Agriculture, Ningxia University, Ningxia 750021, China

## Abstract

A hand-held Raman spectroscopic device was used as a rapid nondestructive testing device to predict the metmyoglobin (MetMb) and metmyoglobin reductase activity (MRA) values on the surface layer of fresh beef. Longissimus dorsi muscles were from 10 young bulls (Holstein-Friesian) from two different cattle farms (group A = 5 and B = 5). The Raman spectra of 100 samples were correlated with the MetMb and MRA values using partial least squares regression (PLSR). Two groups could be discriminated, and the separate correlation models were better than the joint correlation model for the fresh beef. The coefficients of determination are *R*^2^ = 0.81 (group A) and *R*^2^ = 0.87 (group B) for MetMb and *R*^2^ = 0.80 (group A) and *R*^2^ = 0.85 (group B) for MRA. The results show the usefulness of Raman spectra in predicting the inner traits such as MetMb and MRA during meat storage. In conclusion, it is feasible to determine the MetMb and MRA values by Raman spectroscopy. Color is an important indicator of beef freshness and can vary depending on the age, sex, and breed of the cow. They play a very important role in human nutrition. The color of meat is an important indicator of meat freshness, and many researchers are already investigating the causes of color changes. The research was conducted in this environment.

## 1. Introduction

The color is an important indicator of the freshness of beef, which could be changed due to ages, sex, and breeds of cows (Delgado et al., 2002; [21]. They play a very significant part in human diet. The meat color is an important marker of the meat's freshness, and many researches have been done to search for the causes of the color variability. The metmyoglobin accumulation rate is based on the rate of autoxidation of the iron (II) of the protein, myoglobin. Oxymyoglobin, carboxymyoglobin, and metmyoglobin [17] can be used, and metmyoglobin reductase activity is affected [18]; Mikkelsen et al., 1992). Both the nonenzymatic and enzyme reductions have been well studied [22]. Other researchers have studied the metmyoglobin reductase activity of beef with different color stability, and some researchers have found that the MRA of beef with poor color stability is the largest [8]. Though many studies have focused on the key of metmyoglobin reductase in fresh beef meat, the measured method is too time-consuming to be suitable for fast testing. A novel testing method for the metmyoglobin and metmyoglobin reductase activity measurement is rapidly changing this picture.

Raman spectroscopy is one of the technologies that has become more and more popular [2, 3], because it is noninvasive, requires nearly no sample preparation, and is not influenced by the amount water in the sample. The Raman signals come from the inelastic scattering of the incident light from a sample, and the frequency shift of the scattered light shifts in a manner of feature molecular vibrations [14]. Therefore, the Raman spectrum is a vibrational spectrum and may be regarded as a “personal ID” of the scattering material providing quantitative and qualitative information about the molecular structure and composition. Nowadays, the Raman spectroscopy is used as an analytical technique for the evaluation of food quality and safety. In the past, researchers have demonstrated the potential of Raman spectroscopy to predict certain meat quality characteristics such as drip loss, pH, and water holding capacity of pork (Pedersen, Morel, Andersen, & Balling Engelsen, 2003; Scheier, Bauer, & Schmidt, 2014; Scheier, Köhler, & Schmidt, 2014) shear and cooking losses in beef. [2, 25]. Combining chemometric methods with Raman spectroscopy has enabled huge progression for both qualitative and quantitative measurements of food components [27]. Thus, Raman spectroscopy is a nondestructive tool which is potentially suited to predict some meat quality characteristics fast [7].

This is the scattering spectrum. Raman spectroscopy Indian scientist K.V. He invented an analytical method based on the Raman effect to study the scattering spectra of incident light of different frequencies to obtain information about the vibration and rotation of molecules and apply them to the analysis of molecular structure research. Inelastic scattering caused by excitation interactions such as molecular vibrations, optical phonons in solids, and laser light is called Raman scattering. This is an analytical method used to study the molecular structure by analyzing the scattering spectra of light at different frequencies to obtain information about molecular vibrations and rotations.

However, there are no reports on the application of Raman to predict the MetMb and MRA values of fresh beef. The paper reports on a research, which uses 100 samples, to compare whether the measurement of fresh meat by Raman spectra could be used to predict the MetMb and MRA values. In the second part of the paper, the determination of myoglobin reductase activity in the surface layer of fresh beef, Raman spectroscopy processing methods, and processing techniques were described; in the third part, the application of MetMb and MRa values in beef quality identification was analyzed in the spectral processing results; in the fourth part, the parameters of beef quality were analyzed.

## 2. Materials and Methods

### 2.1. Sample Preparation

Longissimus dorsi muscles from 12 young cattle (bulls and cow half, Holstein-Friesian), of approximately 16 months of age, from two different cattle farms (group A = 6 and B = 6, bulls and cow half), Ningxia University, were slaughtered at a commercial slaughter plant (Ningxia Laoheqiao Halal Meat Ware Co. Ltd., China). The mean hot carcass weight was 250.8 ± 25.3 kg (ranging from 225.1 to 297.4 kg). After slaughter, hemoglobin is depleted with blood loss. Then, the color of meat was mainly determined by myoglobin. With the change of myoglobin concentration, the color of the meat change was also obvious. It had a relationship with the spectra [26]. At 24 h postmortem, the meat was cut into slices (20 mm thickness) using a scalpel. And all meat samples were individually vacuum packed and stored at 4°C in the dark. Each group had 50 muscle samples. In there, twenty-five muscle samples were tested without any treatment immediately. The rest of the samples were vacuum packed and stored at 4°C in the dark until preparation on day 5.

24 hours after death, the meat was cut into thin slices with a scalpel. All meat samples were individually vacuum packed and stored at 4°C in the dark. There were 50 muscle samples in each group, and the rest of the samples were discarded. There is no need for direct processing and packaging. Condition at 4°C and store in the dark until day 5 of the preparation date.

### 2.2. Measurement of Metmyoglobin Concentration on the Surface Layer of Fresh Beef

Take the fresh beef topping, mix the fresh beef topping with the fresh beef topping salt buffer solution, and homogenize it with an ultrafine homogenizer and a centrifuge for 20 minutes. The supernatant was filtered through filter paper, and the absorbance of NM was measured at different points with a spectrophotometer. Take the average of the formula to calculate the concentration.

According to the method of Krzywicki et al., [15], a 5 g sample from the surface layer of fresh beef was mixed with 25 mL phosphate buffer (0.04 mol/L, pH = 6.8) and homogenized with an ultrafine homogenizer (FLUKO F6/10, Germany; 10000 rpm, 25 s). After standing for 60 min at 4°C in the dark, the mixture was centrifuged at 4500 × g for 20 min at 4°C. The supernatant was filtered through filter paper, and the absorbance was measured at 525, 545, 565, and 572 nm separately with a spectrophotometer. Each sample was measured three times, and the average value was taken for the further statistical data analysis with the following formula:

(1) $$\begin{array}{c} \text{MetMb}\left(\%\right)=\left(-2.514R1+0.777R2+0.800\text{R}3+1.098\right)\times 100,\\ R1=\frac{A572}{A525},\\ R2=\frac{A565}{A525},\\ R3=\frac{A545}{A525}. \end{array}$$

### 2.3. Measurement of Metmyoglobin Reductase Activity on the Surface Layer of Fresh Beef

Methemoglobin reductase extract was obtained from the skin of fresh beef with minor modifications. Muscle and phosphate-buffered saline were mixed and centrifuged, and fat was removed from the supernatant through a filter paper. Excessive oxidized hemoglobin was added, the solution was dialyzed as a phosphate buffer, and the methemoglobin reductase activity of the extract was measured by spectrophotometry, and the final result was the amount of enzyme activity.

Metmyoglobin reductase extracts were obtained from the surface layer of fresh beef by the Reddy and Carpenter method with slight modifications [22]: 12 g muscle and 20 mL 2.0 mM phosphate buffer (pH = 7.0) were mixed and homogenized with an ULTRA-TURRAX (30 s, 12, 000 rpm, 4°C). The homogenate was centrifuged (30 min, 35000 × g, 4°C), and the fat was removed from the supernatant by a paper filter. Excessive K_3_Fe(CN)_6_ was added to oxidize the oxyhemoproteins, and the solution was dialyzed (14000 MW) against 2 mM phosphate buffer (pH = 7.0). The filtrate was centrifuged (20 min, 15000 × g, 4°C), and the volume of the filtrate was adjusted to 20 mL with 2.0 mM phosphate buffer (pH = 7.0).

Metmyoglobin reductase activity of the extract was measured spectrophotometrically: the standard assay mixture included 0.1 mL water, 0.10 mL 50 mM phosphate buffer (pH = 7.0), 0.10 mL 5.0 mM EDTA (pH = 6.4), 0.1 mL 3.0 mM K_4_Fe(CN)_6_, 0.2 mL 0.75 mM MbFe (III) in 2.0 mM phosphate buffer (pH = 7.0), 0.3 mL extract, and 0.1 mL 2.0 mM NADH. Addition of NADH initiated the reaction. Metmyoglobin reductase activity was calculated as nanomole metmyoglobin reduced per min per g of beef meat during the initial linear phase of the assay, using a difference in molar absorptivity of 12 × 10^3^/mol · cm at 580 nm. The effects of storage (4°C) time of the beef meat prior to extraction as well as the effect of storage time of enzyme extracts were also investigated. Metmyoglobin reductase activities are expressed as the means.

### 2.4. Raman Spectroscopy

**Figure 1:**
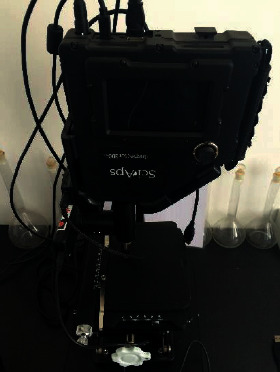
Portable Raman system for on-line measurements of meat consisting of a hand-held Raman device.

An Inspector 300 Raman microscope (SciAps, USA) with a 785 nm laser source, a motorized microscope stage sample holder, and a CCD detector (SciAps, USA) were used. The instrument was used at 10 mW laser power with 8 s acquisition. Each sample was placed on the translation stage and scanned. The spectra were obtained in the range of 400–1700 cm^−1^ at a resolution of 6 cm^−1^ (Figure 1) after calibration using the spectra of polystyrene. Each sample was manipulated with the built-in “automatic baseline correct” function of the software. Five replicate analyses were performed on each sample.

### 2.5. Spectral Processing

In the Raman shift (400–1700 cm^−1^), chemical bonds cause vibrational and rotational transitions, resulting in absorption bands in spectral curves [6]. There are many combination bands that overlap the absorption bands, which make the spectrum highly convoluted and difficult to be interpreted. In addition, other factors, such as the natural light, may also make the spectrum more complex. Therefore, chemometric methods of spectral processing are used to interpret the spectrum. The spectral processing includes spectral pretreatment, modeling, and model evaluation.

In the Raman shift (400–1700 cm^−1^), the compounds induce vibrational and rotational changes, and then, absorption bands appear on the spectral curve, while several combined bands dominate on the d-band. Absorption makes a spectral complex and is difficult to interpret, so spectra can be interpreted using stoichiometric methods of spectral processing.

Raman spectral data across the full wavenumber range were treated by multivariate analysis to find out key information related to the reference MetMb and MRA values. There are a lot of multivariate algorithms for building quantitative models, such as PCA, MLR, and PLS [4]. In this study, PLS and PCR were used to establish predictive models [12]. Prediction of MetMb and MRA values in the models was attained by a group of latent variables, which were statistically uncorrelated and got the most information in *X* (Raman spectral data) and *Y* (reference values of MetMb and MRA values).

Chemical properties of samples and influences in instrument response may cause light scattering effects, and these elements may influence the real responses and the robustness of later multivariate calibration models [24]. In order to decrease or even remove these undesirable elements, pretreatment of the spectral data is necessary, such as multiplicative scatter correction, smoothing, and standard normal variate [20]. In this study, there are four spectral pretreatment methods (Savitzky-Golay smoothing, derivatives, MSC, and Nor). The optimal pretreatment method was chosen by comparing the best performance.

Multivariate data analysis was carried out to predict MetMb and MRA values of samples using their corresponding spectral information. The same Raman spectral dataset can be used together with MetMb and MRA values to build a predictive partial PLS model. Thus, the measured spectra can be used to predicted MetMb and MRA values for the new sample directly. PLSs were carried out to perform linear models of prediction between spectral data and the values of one of the quality parameters obtained from the traditional measurement.

The accuracy of the PLS model was identified based on the coefficient of determination in calibration (*R*^2^_C_), coefficient of determination in crossvalidation (*R*^2^_CV_), root mean square error of calibration (RMSE_C_), and root mean square error by crossvalidation (RMSE_CV_). Usually, a useful model should have high values of *R*^2^ and low values of 4.

### 2.6. Statistical Analysis

The spectral data and computation analyses were operated and completed with the aid of chemometric software MATLABR2011a (The MathWorks Inc., Natick, MA, USA) and Unscrambler X 10.3 (CAMO, Trondheim, Norway).

## 3. Results and Discussion

### 3.1. MetMb and MRA Values

The MetMb and MRA values on the surface layer of beef samples determined by a spectrophotometer are listed in Table 1 (groups A and B). Three replicate analyses were performed on each beef sample . There are 100 beef samples in total. Earlier studies have shown that the fresh meat color should keep bright and stable during storage to maximize consumer acceptance [22].

### 3.2. Spectral Processing

The stoichiometric method of spectral processing is used to interpret the spectra. Spectral processing includes preliminary spectral processing, simulation, and model analysis. Raman spectral data across the entire wavenumber range was processed using multivariate analysis to find key information related to MetMb and MRA reference values.

The Raman spectra were normalized by dividing the intensity using integration time and laser power to compare the two groups A and B. Firstly, the PCA method was used to identify the remaining spectra of fat in the data which had not been removed during data collection. Raman spectra with scores associated to the fat pattern over a threshold value were removed before the next step. The threshold was determined repeatedly by decreasing the value and then making a new PCA model with the rest of the data until the Raman fat pattern was removed from the first 4 loading of the PCA model. By this way, 47 of 1038 Raman spectra of group A and 34 of 1015 spectra of group B were removed. To the next step analysis, the beef Raman spectra were normalized to the baseline intensity at 1517 cm^−1^ to make the PCA and PLS models better. For each sample, the rest of the 12–15 different spectra were averaged and preprocessed using four spectral pretreatment methods (Savitzky-Golay smoothing, derivatives, MSC, and Nor) and mean centering.

PCA, principal component analysis, is an unsupervised machine learning algorithm. It is a method of studying multidimensional data structures. It is mainly used to reduce the dimensionality of data. Downsizing can be used to find features that are easier to understand. It speeds up the processing of valuable information from samples and can also be used for visualization (up to 2D) and noise reduction. And the PCA algorithm simplifies the data and does not depend on parameters.

The performance of PLS models based on three spectral pretreatment methods (MSC, Nor, and Savitzky-Golay smoothing) is shown in Table 2. As shown in Table 2, compared with the model of the original spectral data, the detection effect of the model of the processed spectral data is improved more or less, which indicates that the pretreatment methods can eliminate the noise in the spectral data effectively. For the different detection parameters, the pretreatment methods are also different.

Compared with the results of raw spectra (*R*^2^_p_ of 0.885, RMSE_P_ of 1.387), both of the MSC, Nor, and Savitzky-Golay smoothing methods provided some improvement in model robustness because of higher *R*^2^_p_ and lower RMSE_p_. In the models, the Nor-PLSR model showed the highest values: *R*^2^_p_ of 0.916 and RMSE_p_ of 0.983. So, SGS was chosen as the optimal pretreatment method to build the model of the MetMb values. Similarly, the Nor was chosen for the MAR values.

Spectra show typical Raman signals of muscle tissue. The difference between the Raman spectra of the two sites is a 35% higher broadband spectral background for group B. This may be related to the gender of group B, but the sample or environmental factors cannot be omitted.

**Figure 2:**
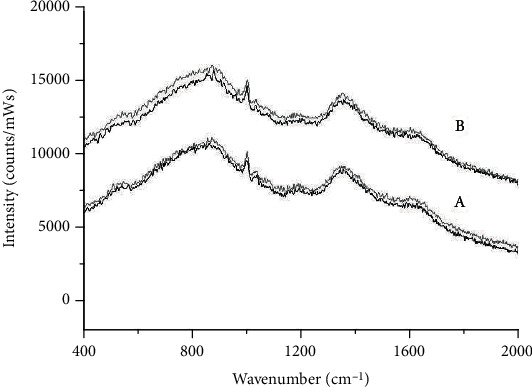
Averaged Raman spectra on the surface layer of fresh beef for A samples and B samples grouped according to their storage time, 0 d (gray curves) and 5 d (black curves).

The samples were put into two storage groups randomly to find out whether the Raman spectra could distinguish two groups.  Figure 2 shows the averaged raw spectra from all beef samples from groups A (lower curves) and B (upper curves) with storage 0 d (gray curves) and 5 d (black curves). For comparison, the Raman spectra were divided by integration time and laser power. The number of samples per group is as follows: 25 storage 0 d and 25 storage 5 d samples for group A and 25 storage 0 d and 25 storage 5 d samples for group B. The spectra exhibited the typical Raman signals of muscle tissue [23]. The difference between the Raman spectra of both sites is a 35% higher broadband spectral background of group B. That could be due to the sex of group B, but the sample or environment elements cannot be ignored. For example, the oxygen content of the vacuum-packaged beef can lead to a variation of the fluorescence level because of the fluorescence quenching effect [16]. So, to reduce the minimum bias, discharge the air as much as possible prior to the Raman measurement. An off-set of the spectral background is also shown between the different storage groups (0 d and 5 d) (Figure 2).

Raw spectra of beef samples were background corrected, showing changes and intensities of protein vibrations (Table 3) at around 930, 1006, 1019, 1230, 1360, and 1655 cm^−1^. The Raman spectra can show the typical Raman signals of muscle [23]. Hydroxyproline, a typical component of connective tissue, may show a signal at 877 cm-1, so the corresponding moderately strong band in connective tissue is not found in the amide III region and is removed [5, 11, 13]. The Raman spectra of the samples seemed to be similar; however, there were spectral data differences due to intensity differences in the same band intensity. The band positioned at 1265 cm^−1^ belongs to *δ*(C-H) bending at the cis double bond in R-HC=CH-R [9]. The bands at 800–900 and 1000–1100 cm^−1^ are due to the vibration of skeletal C-C bonds in −(CH2)n− molecules [1]. The band at 1213 cm^−1^ is due to antisymmetric phosphoryl stretching corresponding to the vibrations of fatty acid and phospholipid chains [10]. Lu (2014) determined the relative amounts of hemoglobin by Raman spectral and found that the spectral differences between the three hemoglobin derivatives were exhibited in several relevant peaks in 1210–1230 cm^−1^, 1375–1379 cm^−1^, and 1550–1650 cm^−1^. Compared to oxyhemoglobin, a number of changes were apparent in the range of 1550–1650 cm^−1^ in the Raman spectra of carboxyhemoglobin. Compared to oxyhemoglobin, in the spectra of carboxyhemoglobin, the band at 1559 cm^−1^ appears to be more intense, while the 1589 cm^−1^ and 1645 cm^−1^ bands were less intense. In the spectra of methemoglobin, a number of changes were exhibited in the range of 1210–1230 cm^−1^, 1375–1379 cm^−1^, and 1550–1650 cm^−1^, by comparing with oxyhemoglobin (Lu et al. 2013). Along with the spectral differences, the peaks show signals of structural traits that can help in the Raman detection of MetMb and MRA. The result show that Raman spectroscopy could be a useful method instead of the time-consuming and destructive mechanical method in the measurement of MetMb and MRA in fresh beef.

**Figure 3:**
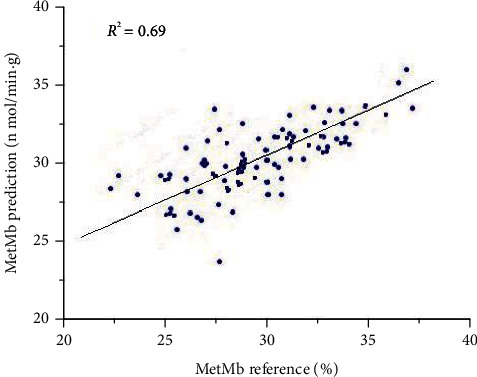
PLS correlation model of Raman data for MetMb on the surface layer of fresh beef and coefficient of determination.

The analysis of A and B groups of the PLS model is showed in Figure 3. In the PLS model, the correlation is moderate with a coefficient of determination of *R*^2^ = 0.79, a root mean square error of calibration (RMSE_C_) of 1.493, and a root mean square error of validation (RMSE_CV_) of 1.203. Further analysis was showed for A and B groups.

**Figure 4:**
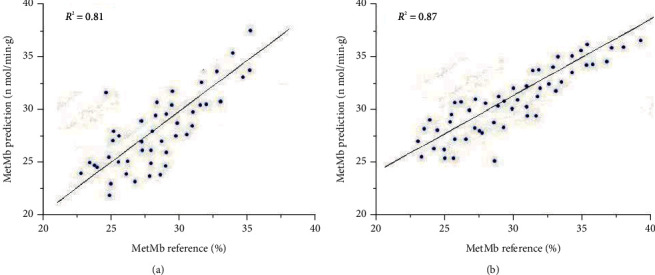
PLS correlation models of Raman data for MetMb on the surface layer of fresh beef and coefficient of determination((a) A samples; (b)B samples).

PLS regression analysis correlating the Raman spectra with the MetMb values for the subsets of samples shows a coefficient of determination of *R*^2^ = 0.81 (A) and *R*^2^ = 0.87 (B). The result of the analysis is showed in Figure 4. The RMSE_CS_ are computed to be 1.276 (A) and 1.218 (B). It can evaluate the validity of the PLS model. The values were not satisfactory because of the MetMb values being distributed unevenly, i.e., rather few samples with low MetMb values were measured. The more evenly distributed of MetMb data making the coefficient of determination was better than the A subset.

**Figure 5:**
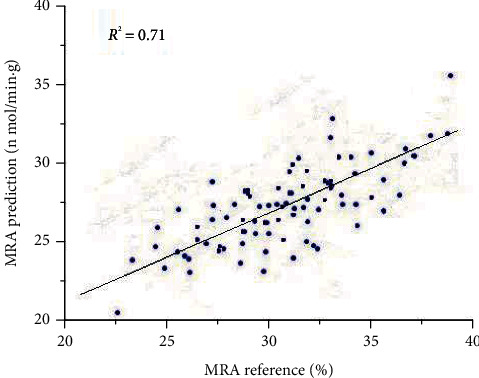
PLS correlation model of Raman data for MRA on the surface layer of fresh beef and coefficient of determination.

The Raman spectra was correlated with the MRA reference measurements. The joint treatment of A and B subsets brought about a less good enough model (Figure 5). The coefficient of determination of *R*^2^ = 0.71, RMSE_C_ = 1.626%, and RMSE_CV_ = 1.395 yield reasonable values for the model, respectively.

The distribution of MetMb values is not the same, i.e., few models with low MetMb values are measured, so these values are not satisfactory. The MetMb data distribution of the coefficient of determination is better than that of subset A. The Raman spectrum is related to the ARM reference measurement, and the coefficients for each setting provide reasonable values for the model.

**Figure 6:**
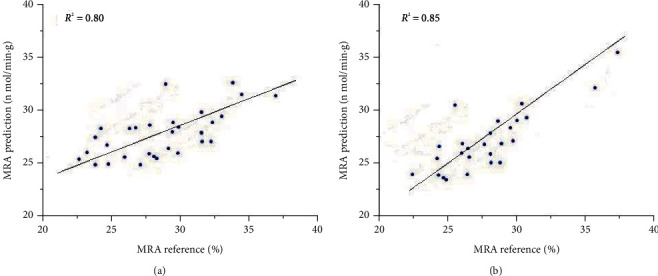
PLS correlation models of Raman data for MRA on the surface layer of fresh beef and coefficient of determination((a) A samples; (b) B samples).

The separate treatment of the A and B groups improves the results (Figure 6). *R*^2^ = 0.80 for A samples and *R*^2^ = 0.85 for B samples. RMSE_C_ = 1.193 and RMSE_CV_ = 0.947 for A samples and RMSE_C_ = 1.063 and RMSE_CV_ = 0.802 for B samples. Therefore, for the MetMb measurements, the correlation of the B group was slightly better than that of the A group. To be used in meat production accurately and widely, the samples must be more diverse and also have strong predictions of the characteristics of measurement after a period of storage. These problems will be studied in the future research.

## 4. Conclusion

PLS regression uses the principle of principal component analysis to combine multiple *X* and multiple *Y* components (*X* is the principal component of *U*, *Y* is the principal component of *V*) and then uses the principle of canonical correlation to determine the relationship between *X* to analyze the *Y* relationship between *X* and *V*, combined with the principle of multiple linear regression, analyze the relationship between *X* and *V*, and explore the relationship between *X* and *Y*.

The Raman spectra of samples of two different cattle farms A and B could be correlated with the values of MetMb and MRA on the surface layer of fresh beef using PLS regression analysis. Groups and B group could be significantly distinguished by the different Raman spectra. And the distinction is based on spectral broadband differences. But the reasons of the broadband differences was not unclear. In the Raman spectra of beef samples in different storage time and sources, it can recognize signals that come from *α*-helical protein structures, the aromatic amino acid side chain of tryptophan and phenylalanine. The correlation models of separate correlation of Raman spectra subsets A and B were better than the joint correlation for the all data. The result show that Raman spectroscopy could be a useful method instead of the time-consuming and destructive mechanical method for the prediction of quality traits such as MetMb and MRA values in fresh beef meat. The future study should research the prediction ability of Raman spectra for the quality traits of fresh beef when the spectra are collected on meat during the full storage time. Considering the application of new technologies in the future, intelligent computing combined with the PLSR algorithm is used for parameter optimization and application. Further, the beef quality of different varieties was classified and the key factors of beef quality were discussed and analyzed in depth under the influence of various factors.

## Figures and Tables

**Table 1:** Mean ± s.d., minimum and maximum for the measured traits: carcass weight (kg), MetMb (%), and MAR (nmol/min·g) according to the group (50 samples per group).

	Group	Minimum	Maximum	Mean ± s.d.
MetMb (%)	A	13.52	31.08	22.30 ± 4.78
	B	16.95	38.16	27.56 ± 5.61
MRA (nmol/min·g)	A	10.48	29.08	19.78 ± 3.72
	B	9.73	31.26	20.49 ± 4.99

**Table 2:** PLS models for predicting MetMb and MRA values on the surface layer of fresh beef with raw and pretreated spectral data.

	Pretreatment	Number of latant number	Calibration		Crossvalidation		Prediction	
			*R* ^2^_c_	RMSE_c_	*R* ^2^_cv_	RMSE_cv_	*R* ^2^_p_	RMSE_p_
MetMb	Raw	6	0.827	1.169	0.804	0.985	0.885	1.387
	SGS	5	0.856	0.815	0.832	0.693	0.916	0.983
	MSC	6	0.837	0.911	0.815	0.741	0.908	1.157
	Nor	5	0.846	0.894	0.797	0.733	0.911	0.9981
MAR	Raw	4	0.762	1.416	0.801	0.857	0.806	1.514
	SGS	5	0.801	1.325	0.853	1.215	0.845	1.502
	MSC	6	0.818	0.993	0.862	0.819	0.853	1.361
	Nor	6	0.836	0.869	0.887	0.786	0.871	0.908

**Table 3:** Raman modes useful in interpretation of the protein structure adapted from the works of Li-Chan [19] and Beattie et al. [3].

Origin	Wavenumber (cm^−1^)	Assignment	Structural information obtained
Phenylalanine	1006	Breathing ring	Conformation insensitive; useful as an internal intensity standard
Histidine	1409	N-Diimidazole	Probe of ionization state, metalloprotein structure, and proton transfer in deuterated solution
Tryptophan	760, 880, 1360	Indole ring	Sharp intense band indicates buried residues; sensitive to environment polarity
Aspartic acid, glutamic acid	1400–1430	C=O stretch of COO−	Ionized carboxyl groups
Amide III	1230–1240	N-H in-plane bend, C-N stretch	Antiparallel g-sheet
Amide I	1650–1660	Amide C=O stretch, N-H wag	*α*-Helix
Aliphatic amino acids	1450, 1465	C-H bending	Microenvironment, polarity

## Data Availability

The experimental data used to support the findings of this study are available from the corresponding author upon request.

## References

[1] Baeten V., Hourant P., Morales M. T., Aparicio R. (1998). Oil and fat classification by FT-Raman spectroscopy. *Journal of Agricultural and Food Chemistry*.

[2] Beattie R. J., Bell S. J., Farmer L. J., Moss B. W., Patterson D. (2004). Preliminary investigation of the application of Raman spectroscopy to the prediction of the sensory quality of beef silverside. *Meat Science*.

[3] Beattie J. R., Bell S. J., Borggaard C., Fearon A., Moss B. (2007). Classification of adipose tissue species using Raman spectroscopy. *Lipids*.

[4] Blanco P., Sieiro C., Villa T. G. (1999). Production of pectic enzymes in yeasts. *FEMS Microbiology Letters*.

[5] Carew E. B., Stanley H. E., Seidel J. C., Gergely J. (1983). Studies of myosin and its proteolytic fragments by laser Raman spectroscopy. *Biophysical Journal*.

[6] Cen H., He Y. (2007). Theory and application of near infrared reflectance spectroscopy in determination of food quality. *Trends in Food Science and Technology*.

[7] Damez J. L., Clerjon S. (2008). Meat quality assessment using biophysical methods related to meat structure. *Meat Science*.

[8] Michardiere R., Salem D. B. (2021). Comparison of MRA and arteriography in the monitoring of intracranial aneurysms treated with gdc. *Journal of Neuroradiology*.

[9] El-Abassy R., Eravuchira P., Donfack P., Von der Kammer B., Materny A. (2011). Fast determination of milk fat content using Raman spectroscopy. *Vibrational Spectroscopy*.

[10] Gallier S., Gordon K. C., Jiménez-Flores R., Everett D. W. (2011). Composition of bovine milk fat globules by confocal Raman microscopy. *International Dairy Journal*.

[11] Herrero A. M. (2008). Raman spectroscopy for monitoring protein structure in muscle food systems. *Critical Reviews in Food Science and Nutrition*.

[12] He H.-J., Wu D., Sun D.-W. (2014). Potential of hyperspectral imaging combined with chemometric analysis for assessing and visualising tenderness distribution in raw farmed salmon fillets. *Journal of Food Engineering*.

[13] Ikoma T., Kobayashi H., Tanaka J., Walsh D., Mann S. (2003). Physical properties of type I collagen extracted from fish scales of Pagrus major and Oreochromis niloticas. *International Journal of Biological Macromolecules*.

[14] Kneipp K., Kneipp H., Itzkan I., Dasari R. R., Feld M. S. (1999). Ultrasensitive chemical analysis by Raman spectroscopy. *Chemical Reviews*.

[15] Krzywicki K. (1982). The determination of haem pigments in meat. *Meat Science*.

[16] Lakowicz J. R., Weber G. (1973). Quenching of protein fluorescence by oxygen. Detection of structural fluctuations in proteins on the nanosecond time scale. *Biochemistry*.

[17] Ledward D. A. (1992). Colour of raw and cooked meat. *Special Publication-Royal Society of Chemistry*.

[18] Chen Y., Cheng H., Tram K. (2013). A paper-based surface-enhanced resonance Raman spectroscopic (SERRS) immunoassay using magnetic separation and enzyme-catalyzed reaction. *Analyst*.

[19] Li-Chan E. C. Y. (1996). The applications of Raman spectroscopy in food science. *Trends in Food Science & Technology*.

[20] Luypaert J., Heuerding S., Heyden Y. V., Massart D. L. (2004). The effect of preprocessing methods in reducing interfering variability from near-infrared measurements of creams. *Journal of Pharmaceutical and Biomedical Analysis*.

[21] McAfee A. J., McSorley E. M., Cuskelly G. J. (2010). Red meat consumption: an overview of the risks and benefits. *Meat Science*.

[22] Mikkelsen A., Juncher D., Skibsted L. H. (1999). Metmyoglobin reductase activity in porcine m. longissimus dorsi muscle. *Meat Science*.

[23] Pezolet M., Pigeon-Gosselin M., Caille J. P. (1978). Laser Raman investigation of intact single muscle fibers. *Biochimica et Biophysica Acta (BBA)-Protein Structure*.

[24] Rinnan A., Berg F. V. D., Engelsen S. B. (2009). Review of the most common preprocessing techniques for near-infrared spectra. *TrAC Trends in Analytical Chemistry.*.

[25] Schmidt H., Scheier R., Hopkins D. L. (2013). Preliminary investigation on the relationship of Raman spectra of sheep meat with shear force and cooking loss. *Meat Science*.

[26] Xiong Z., Sun D. W., Pu H., Xie A., Han Z., Luo M. (2015). Non-destructive prediction of thiobarbituricacid reactive substances (TBARS) value for freshness evaluation of chicken meat using hyperspectral imaging. *Food Chemistry*.

[27] Yang H., Irudayaraj J., Paradkar M. M. (2005). Discriminant analysis of edible oils and fats by FTIR, FT-NIR and FT-Raman spectroscopy. *Food Chemistry*.

